# Spatial structure in migration routes maintained despite regional convergence among eastern populations of Swainson’s Thrushes

**DOI:** 10.1186/s40462-021-00263-9

**Published:** 2021-05-13

**Authors:** Camille Bégin-Marchand, André Desrochers, Philip D. Taylor, Junior A. Tremblay, Lucas Berrigan, Barbara Frei, Ana Morales, Greg W. Mitchell

**Affiliations:** 1 Wildlife Research Division, Environment and Climate Change Canada, 1550 d’Estimauville, Québec, QC G1J 0C3 Canada; 2 Université Laval, 2325 Rue de l’Université, Québec, QC G1V 0A6 Canada; 3 Acadia University, 33 Westwood Avenue, Wolfville, NS B4P 2R6 Canada; 4 Birds Canada, P.O. Box 6227, 17 Waterfowl Lane, Sackville, NB E4L 1G6 Canada; 5 McGill Bird Observatory, The Migration Research Foundation, Inc., PO Box 10005, Ste Anne de Bellevue, QC H9X 0A6 Canada; 6 Canadian Wildlife Service, Environment and Climate Change Canada, 351 boul. Saint-Joseph, Gatineau, QC K1A 0H3 Canada; 7 McGill University, 21111 Lakeshore Road, Ste., Anne de Bellevue, QC H9X 3V9 Canada; 8 Wildlife Research Division, Environment and Climate Change Canada, 1125 Colonel By Rd., Ottawa, ON K1A 0H3 Canada; 9 Department of Biology, Carleton University, 1125 Colonel By Dr., Ottawa, ON K1S 5B6 Canada

**Keywords:** Migration pace, Migratory connectivity, Motus, Neotropical migrants, Radio-telemetry

## Abstract

**Background:**

Migratory connectivity links the different populations across the full cycle and across the species range and may lead to differences in survival among populations. Studies on spatial and temporal migratory connectivity along migration routes are rare, especially for small migratory animals.

**Methods:**

We used an automated radio-telemetry array to assess migratory connectivity *en route* and between early and later stages of the fall migration of the eastern populations of Swainson’s Thrush, and to assess the variation of migration pace between consecutive detection from the different receiving stations along the migratory journey. We tracked 241 individuals from across eastern Canada to determine if populations were mixing around the Gulf of Mexico. We also tested the influence of tagging longitude, latitude and age on migration pace.

**Results:**

Migration routes varied and converged towards the northeast coast of the Gulf of Mexico, but in this region, populations maintained finer-scale spatial structure. Migration pace increased as birds progressed south, independent of age and tagging site.

**Conclusions:**

We showed that for songbirds, migratory connectivity can be maintained at fine spatial scales despite the regional convergence of populations, highlighting the importance of detailed spatial tracking for identification of population specific migration routes. Overall, our study provides a portrait of migratory movements of eastern Swainson’s Thrush and a framework for understanding spatial structure in migration routes for other species.

**Supplementary Information:**

The online version contains supplementary material available at 10.1186/s40462-021-00263-9.

## Background

Migratory connectivity describes the movements and locations of migratory birds across seasons. More precisely, it describes where and when different populations of a species do or do not converge during the full annual cycle [55]. Research on migratory connectivity often limits its scope to the breeding and wintering periods [14]. Studies linking breeding populations to specific geographic locations along migration routes are rarer [29, 50], particularly for small migratory animals. The strength of migratory connectivity between the breeding grounds and along migration routes is likely driven by both proximate and ultimate drivers, including geographic breeding origin, age, sex, body condition and evolutionary history [44, 47, 8, 50]. Although individuals from populations with broad longitudinal breeding distributions have different migration routes, at least initially, they might converge regionally at important refuelling or resting areas [4, 21, 37]. Importantly, it is unclear whether birds maintain their original population spatial structure at finer spatial scales within regions when they aggregate in specific migratory routes or stopover sites. Furthermore, another important aspect of migratory connectivity that is currently understudied is the temporal linkage of individuals, i.e. whether the individuals of the same species are found at a specific stopover site during the same period of time. Understanding the spatial and temporal migratory connectivity of populations during migration can help identify “bottlenecks” that are most likely to affect the survival of entire breeding populations, or alternatively, sub-populations [20, 29, 37, 55].

The Swainson’s Thrush (*Catharus ustulatus*) is a long-distance migrant whose breeding range extends from western to eastern Canada and portions of the northern United States [33, 51]. Migratory connectivity has been shown to be very strong with respect to migration routes for western populations of inland (*C. u. ustulatus*) and coastal (*C. u. swainsoni*) Swainson’s thrush [16, 28, 47]. However, differences among eastern populations with respect to migration routes are less clear [5, 30]. Banding data [8], stable isotope analysis [8], genetic markers [47, 8] and citizen scientist observations [30, 51] all suggest that eastern populations of Neotropical migrants, including Swainson’s Thrush, overwinter in Central America and northern South America, and likely migrate along an eastern migration route converging in the southeastern states, north of the Gulf of Mexico. However, the accuracy of the migratory movements currently described are not sufficient to assess migratory connectivity at finer spatial scales during the migration period.

The miniaturization of tracking devices and the development of automated telemetry networks is now allowing researchers to track migratory movements of small animals at both broad and fine spatial scales [35, 36, 45, 52]. The Motus network is a collaborative radio-telemetry array distributed mainly across the American continent, and mostly in eastern North America [52]. In the last decade, Motus has been used to study movement behavior at regional and continental scales [3, 5, 10, 27, 48]. While geolocators and other archival tags have revolutionized our understanding of migration routes and timing, unfortunately, they only provide information on individuals that survived their entire migration, potentially biasing our understanding of migration routes to highly philopatric and high quality individuals or individuals that took the safest/best migration routes. Automated telemetry networks on the other hand, such as Motus, provide at least some tracking information on almost all individuals tagged and currently offers finer-scale spatial tracking information.

Our overall objective was to evaluate the spatial and temporal components of migratory connectivity during the migration period for eastern populations of Swainson’s thrushes and evaluate whether the individuals maintained their original population structure throughout their migration routes. Based on other studies using various intrinsic and extrinsic markers or citizen science (described above), we expected that migratory connectivity would decrease as the birds approach the Gulf of Mexico, i.e. that migration routes would initially differ along a longitudinal gradient and later converge near the Florida peninsula [30, 37]. We also expected that migration pace would slow as birds approached the Gulf of Mexico in the southern United States as they potentially stopover for longer periods to fuel in preparation to undertake an overwater flight to reach their wintering grounds [4, 5, 37]. We also predicted that, regardless of tagging site, adult birds would migrate at a faster pace than juvenile birds given previous knowledge of migration routes and a propensity for adults to have a better foraging proficiency [59] and to select more favorable weather conditions for migration relative to juvenile birds [15, 39, 58].

## Methods

### Study sites and radio-telemetry array

We gathered data on 392 individuals from six bird observatories and research stations distributed within the northeastern range of Swainson’s Thrush that were fitted with Motus tags between 2014 and 2018. Data from the eastern Great Lakes (Bruce Peninsula Bird Observatory, hereafter ‘BPBO’: 45.25, − 81.30), southwestern Quebec (McGill Bird Observatory, hereafter ‘MBO’: 45.43, − 73.94), southeastern Quebec (Parc national des Monts-Valin, hereafter ‘MV’, 48.61, − 70.83 and Forêt Montmorency, hereafter ‘FM’, 47.37, − 71.10), the Quebec-Labrador peninsula (Observatoire d’Oiseaux de Tadoussac, hereafter ‘OOT’, 48.16, − 70.83) and the Maritimes (Atlantic Bird Observatory, hereafter ‘ABO’, 43.45, − 65.82) were included in the analysis. Individuals were captured during fall migration (BPBO, MBO, OOT) or directly on their breeding site (FM, MV, ABO). We used different Lotek Avian nanotag models (Lotek, Newmarket, ON) with distinct burst intervals and estimated lifespan across years and sites of capture (see Supplementary material: Table S2). Together, the nanotag and harness weighed less than 4% of the mean body mass of all captured individuals. This mass has been shown not to affect the migration behavior of other *Catharus* species [43, 54]. We removed false detections due to random noise or static near receiving stations within the radio-telemetry array, following the method of Crewe et al. [60]. We retained detections from the beginning of August to the end of November, between the tagging site and the tip of the Florida peninsula, and excluded detections within 100 km from the tagging site to test for fall migratory movements. Birds captured on their breeding site at the ABO were surrounded by a high concentration of receiving stations and Swainson’s Thrush are known to engage in extensive post-breeding movements in this area [6], thus we removed detections within 300 km of the tagging site to exclude post-breeding movements [6].

### Differences in migration routes

We estimated (1) spatial migratory connectivity between early and later stages of migration and (2) spatial migratory connectivity *en route*, as well as (3) temporal migratory connectivity *en route*. Migratory connectivity can be estimated by performing a Mantel test [1, 14, 31], which consists of testing the correlation between distance matrices from individuals at the origin site to the final destination. The resulting *R*_*m*_ varies between 0 (absence of migratory connectivity among populations) and 1 (strong migratory connectivity, i.e. complete spatial segregation of the different populations).

To compare spatial migratory connectivity between the early and later stages of migration in Canada and the U.S., we calculated the geographic distance between individuals at their tagging location (i.e. near or on their breeding site) and the geographic distance between individuals at their last detection [1, 14]. Only individuals with at least one detection south of 30°N (south of northern Florida) were included in this analysis. We tested the correlation between distances and performed a Mantel test using 10,000 permutations to determine whether distance between individuals at tagging locations were correlated with distances between individuals near the Florida peninsula. Analysis were performed with the R package *MigConnectivity* [14].

In addition to evaluating migratory connectivity between the early and later stages of migration, we also evaluated migratory connectivity *en route* using a different approach than that described above (see [5]). Importantly, while the method described above evaluates connectivity based on correlations of distances between populations at two points in time, the method described below accounts for where animals are located geographically in space during migration. This is important, because despite a potential lack of correlation in distances between individuals from different populations, there still may be spatial structure present. Consider two examples. First, there could be situation where migratory connectivity is maintained longitudinally, but differences in location latitude at final locations mask this spatial structure when only evaluated using a distance approach. Second, there could be a situation where migration routes cross each other, but where longitudinal spatial structure (migratory connectivity) is maintained. Again, a distance based approach for derivation connectivity may mask this spatial structure.

To evaluate migratory connectivity *en route*, we aggregated receiving stations into latitude-longitude cells at 3 different degree scales (0.01 × 0.01, 0.1 × 0.1 and 1 × 1 degree) and noted the presence/absence of every individual for every cell [5]. We built a matrix (individual x individual) to calculate a Bray-Curtis dissimilarity index (0 = individuals are detected at the same cells, 1 = individuals are detected at a completely different set of cells) [23] with the R package *vegan* [42]. For this analysis, we performed the partial Mantel test [31, 32, 49] to determine the correlation between the individual matrix and the tagging location, and to control for year of capture, as the number and position of receiving stations varied between 2014 and 2018. To estimate the variation of migratory connectivity *en route*, i.e. at different stages during the migratory journey between tagging location and southeastern U.S, we reproduced the analysis for 16 intervals of 5°latitude between 45°N and 25°N, shifted by one degree at each interval (e.g.45–31°N, 44–30°N, 43–29°N, …, 29–25.). We performed 10,000 permutations for each partial Mantel test [31] and calculated a 95% confidence interval based on 100 bootstrap samples (see Supplementary material: Table S1 for more information on the result of each interval). To evaluate the temporal migratory connectivity, we used the same method described above used for spatial migratory connectivity *en route* but we used the Julian dates of detection within the latitude interval in of latitude-longitude cells [29].

Last, to further evaluate the spatial structure of the last detections in northern Florida and provide more spatial context to our results from above, we also fitted a multivariate multiple regression model of the last detection latitude and longitude as a function of tagging longitude and year of capture.

### Differences in migration pace

The radio-telemetry receiving array did not allow us to calculate exact in flight ground speeds because detection range and distance between receiving stations were variable. In addition, we cannot assume that movements between receiving stations were linear. The farther apart the receiving stations are from one another, the less we can infer the bird’s behavior between them (stopover duration and number, average flight speed, distance travelled, etc.). Thus, we calculated, based on sequential detections between two receiving stations (hereafter ‘segments’), a migration pace (km/h) using the distance between receiving stations and the time elapsed between the reception of the strongest signal at each receiving station within an hour [5, 27]. Thus, we limited the underestimation of the distance travelled due to the variation of the detection range among the different receiving stations [52]. To exclude local movements during stopover [38, 53] and simultaneous detections that result in unrealistically high migration pace measures, we calculated migration pace between detections a minimum of 30 min apart and from two different receiving stations located a minimum of 30 km apart. For each segment, we calculated the midpoint between both receiving stations to assess the detection latitude and longitude for each segment. Given that density of the telemetry network is highly variable among regions, bias might be introduced into our measurement of migration pace. For example, stationary periods like resting or stopover will lower migration pace to a greater extent when receiving stations are nearby [5]. To illustrate this, consider one hypothetical bird flying at a constant ground speed of 50 km/h which makes an 8-h stop along a 100 km segment between two stations, yielding a migration pace of 10 km/h. Now consider another bird with the same ground speed making a 30-h stop along a 1000 km segment, yielding at a migration pace 20 km/h. The latter pace is twice as high, despite a stopover lasting more than three times that of the first bird. Thus, the interpretation of the migration pace to determine stopover or stationary periods depends on the distance travelled and not only on the resulting migration pace itself as a 8-h stopover period likely has a different ecological function than a staging period > 24-h [4, 37]. Furthermore, birds are more likely to exhibit stationary periods as the distance travelled increases, which would have the effect of decreasing migration pace. Considering those two biases, we included the distance between receiving stations as a covariate.

To analyze migration pace data we built four GAMMs with the R package *mgcv* [56] to test which variables, including detection latitude (midpoint), tagging longitude, age (juveniles vs adults) and the interaction between age and latitude had an effect on migration pace (log) (see Table 1 for details on model specification). We included year of capture (2014 to 2018) and bird ID as random effects. We compared each of the four models using second-order Akaike’s Information Criterion adjusted for small sample sizes (AICc )[26]. AICc is a measure of model performance, which compares the maximum likelihood estimates of the models, while penalizing for increasing complexity. Models were ranked according to the strength of support for each model, using measures of the difference between each candidate model and the most informative model (with the lowest AICc) [2]. AICc values were derived using the *MuMIn* package [61]. As with our model of migration longitude, we visually assessed residuals plot of the global model and our best-fitting model (lowest AICc) to assess model fit. We performed model averaging on all four models to test for the influence of each variable included in our competing models on migration pace.

**Table 1:** Selection of 4 generalized additive models (GAMs) to describe the influence of latitude, tagging longitude, age and the interaction between age and latitude on migration pace (log) and their relative weight according to Akaike’s criterion. Bird ID and year of capture were random effects (re) and distance between receiving stations was included as a covariate. The strongest model (bold) is the full model and the fourth is the null model

Model	AIC	Delta AIC	Model likelihood	AIC weight
**s(tagging longitude) + s(latitude) + age + s(latitude, by = age, m = 1) + s(distance) + s(year(re)) + s(bird ID (re))**	**1898.97**	**0.0**	**1.0**	**0.56**
s(tagging longitude) + s(latitude) + s(distance) + s(year(re)) + s(bird ID (re))	1899.72	0.75	0.69	0.39
s(latitude, by = age, m = 1) + age + s(distance) + s(year(re)) + s(bird ID (re))	1903.75	4.78	0.09	0.05
s(distance) + s(year(re)) + s(bird ID (re))	1908.13	9.15	0.01	0.01

## Results

### Migration routes of eastern Swainson’s Thrush

**Fig. 1:**
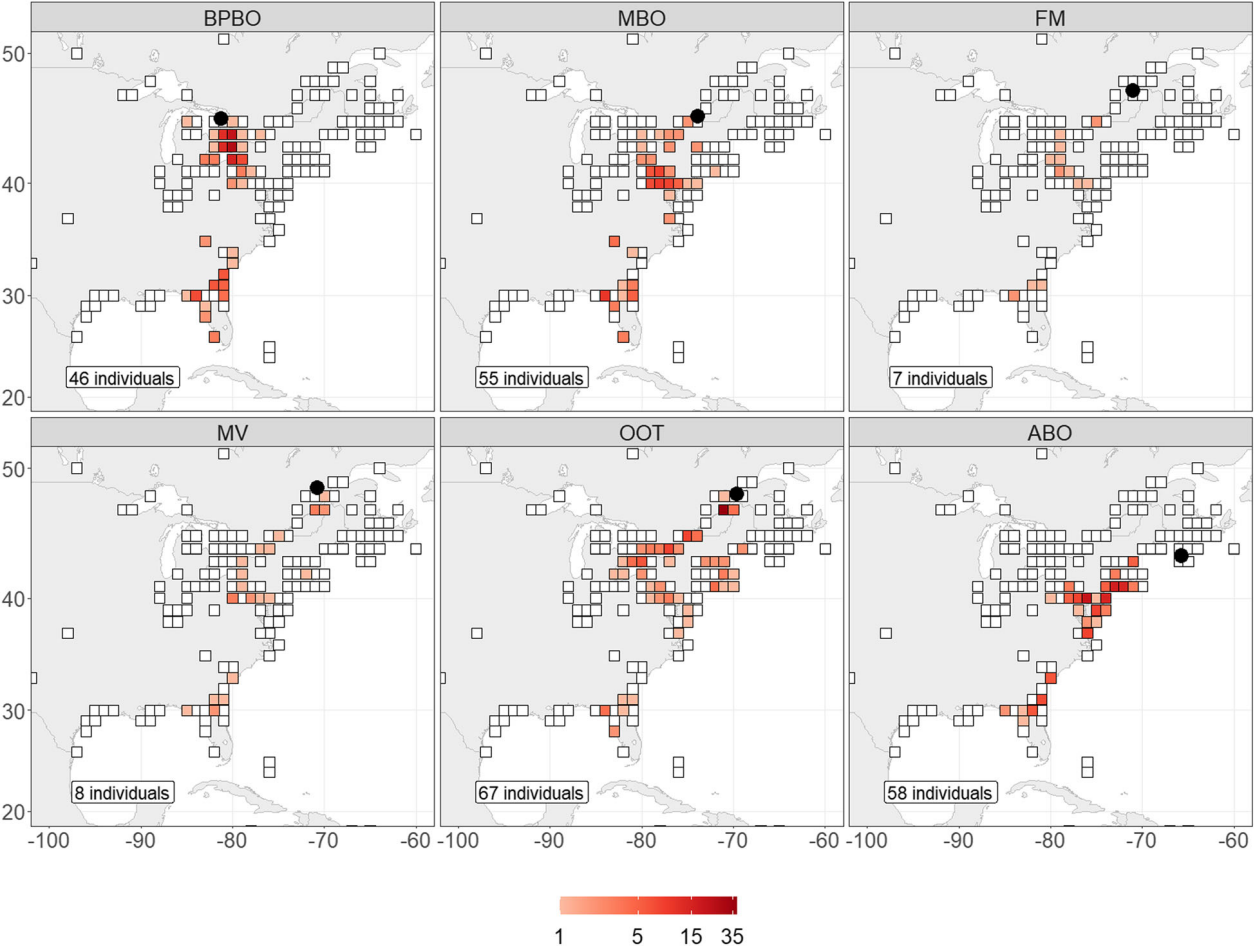
Number of birds detected in the Motus network from different tagging locations (solid black circles). Receiving stations were aggregated in cells of 1 × 1 degree. Every individual was counted only once per receiving station. Empty cells are receiving stations with no detections and colored cells represent the number of individuals detected per cell

Of 391 Swainson’s Thrushes tagged in eastern Canada between 2014 and 2018, we obtained 567 detections (n) for 241 individuals during their fall migration (detected/tagged): eastern Great Lakes (BPBO, 46/49 individuals), southwestern Quebec (MBO: 55/76 individuals), southeastern Quebec (MV: 8/8 individuals, and FM: 7/49 individuals), the Quebec-Labrador peninsula (OOT:, 67/102 individuals) and the Maritimes (ABO: 58/108 individuals). The remaining 151 individuals were not detected beyond 100 km from their tagging site (300 km for ABO individuals) and were removed from further analysis. Individuals exhibited a longitudinal gradient divided into different migration routes between the Appalachian Highlands and the Atlantic plains (Fig. 1). More specifically, individuals tagged at both extremes (west: Great Lakes i.e. BPBO, east: Atlantic Canada i.e. ABO) were detected by a completely different set of receiving stations before reaching southerner states.

### Migratory connectivity

**Fig. 2:**
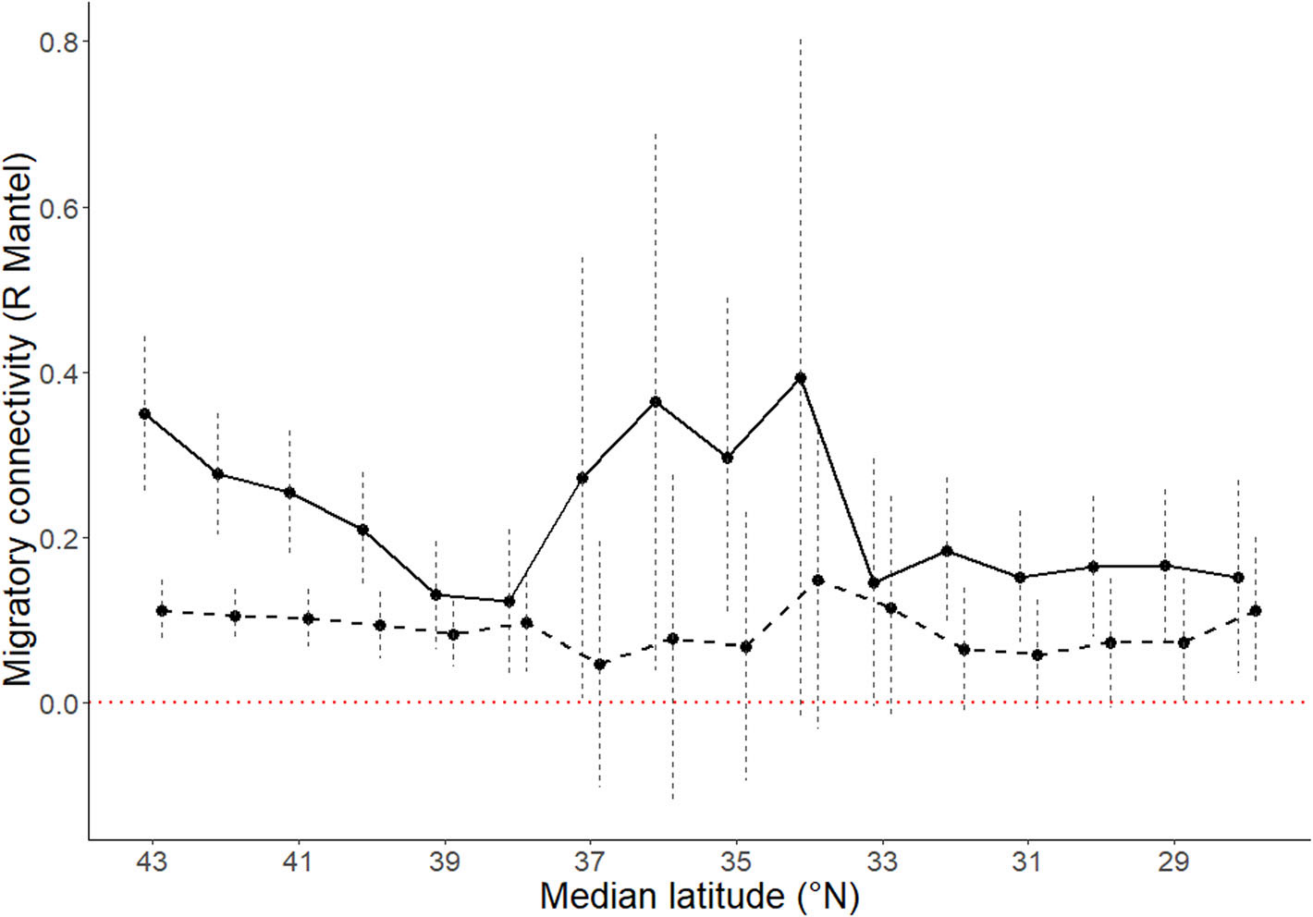
Variation of the spatial (continuous line) and temporal (hatched line) migratory connectivity (Mantel *Rm* statistic) *en route* between tagging sites along a latitudinal gradient of cells of 1 × 1 degree (values close to 0 = weak migratory connectivity, values close to 1 = strong migratory connectivity). Populations converged near the Florida peninsula but maintained a finer scale spatial structure (continuous line). We calculated the median latitude of the 16 intervals of 5°N tested. Observations between 37 and 33 °N did not include enough receiving stations for a robust statistical interpretation. Populations maintained a temporal segregation (hatched line) in the early stages of migration, but no differences associated to the origin was found south of 38°N, despite a slight increase, but weak connectivity, in the last detections south of 30°N. Vertical bars represent 95% confidence limits based on 100 bootstrap samples. The read horizontal line represents the y-intercept = 0

Our migratory connectivity analysis based on distances between tagging locations and distances between final detection locations around Florida was not significant (*r*_*M*_ = 0.04 ± 0.04, 95% CI = [− 0.02, 0.12]). This suggests that migrating Swainson’s Thrushes spatially converged around the Florida peninsula, but that distances between detections at receiving stations around Florida was not dependent of the distances between birds at the beginning of their tracking. Similarly, our assessment of spatial migratory connectivity *en route*, accounting for spatial locations of detections, suggests that migratory connectivity decreased swiftly as individuals progressed south, and stabilized around 33°N, i.e. roughly near Georgia and South Carolina (Fig. 2). However, different from our connectivity analysis based on distance, our assessment of migratory connectivity *en route* also suggested that spatial structure in migration routes was maintained around the Florida peninsula as indicated by a statistically significant dissimilarity index near the Florida peninsula (e.g. Figure 2, latitude interval: [32,27[, *r*_*M*_ = 0.17, 95% CI = [0.08, 0.25]). Observations roughly between southern Virginia and northern Florida did not include enough receiving stations for a robust statistical interpretation, as expressed by the large 95% confidence intervals (Fig. 2). The size of the latitude-longitude squares (0.01, 0.1 and 1 degree) did not change the variation of migratory connectivity along latitude, so we only retained the results for squares of size 1 × 1 degree. More information regarding the number of individuals and detections included in every test (interval) is available in Supplementary material (Table S1).

Our multivariate multiple regression supported the finding from our *en route* analysis, where we found a significant correlation between final detection latitude and tagging location (adjusted *R*^2^ = 0.18, *p* = 0.04), however, we did not find a relationship between the detection longitude and tagging location (adjusted *R*^2^ = − 0.01, *p* = 0.5, *n* = 32). Specifically, birds tagged at more western locations (BPBO) were detected in southern Florida more so than birds from more eastern locations (ABO, (*β* = 0.1 ± 0.04, *p* = 0.02).

Our assessment of temporal migratory connectivity suggests that migratory connectivity was significant, but weak and stable until the birds reached North Carolina (median = 38°N, Fig. 2). Migratory connectivity was not significant during the subsequent migration route, suggesting no differences in the timing of the different populations, but it did increase slightly at more southern latitudes near the Florida peninsula. Overall, are temporal connectivity analysis suggests that the different populations had a different migration timing at the beginning of their route and again, just before crossing or circumventing the Gulf of Mexico.

### Migration pace along a latitudinal gradient

**Fig. 3:**
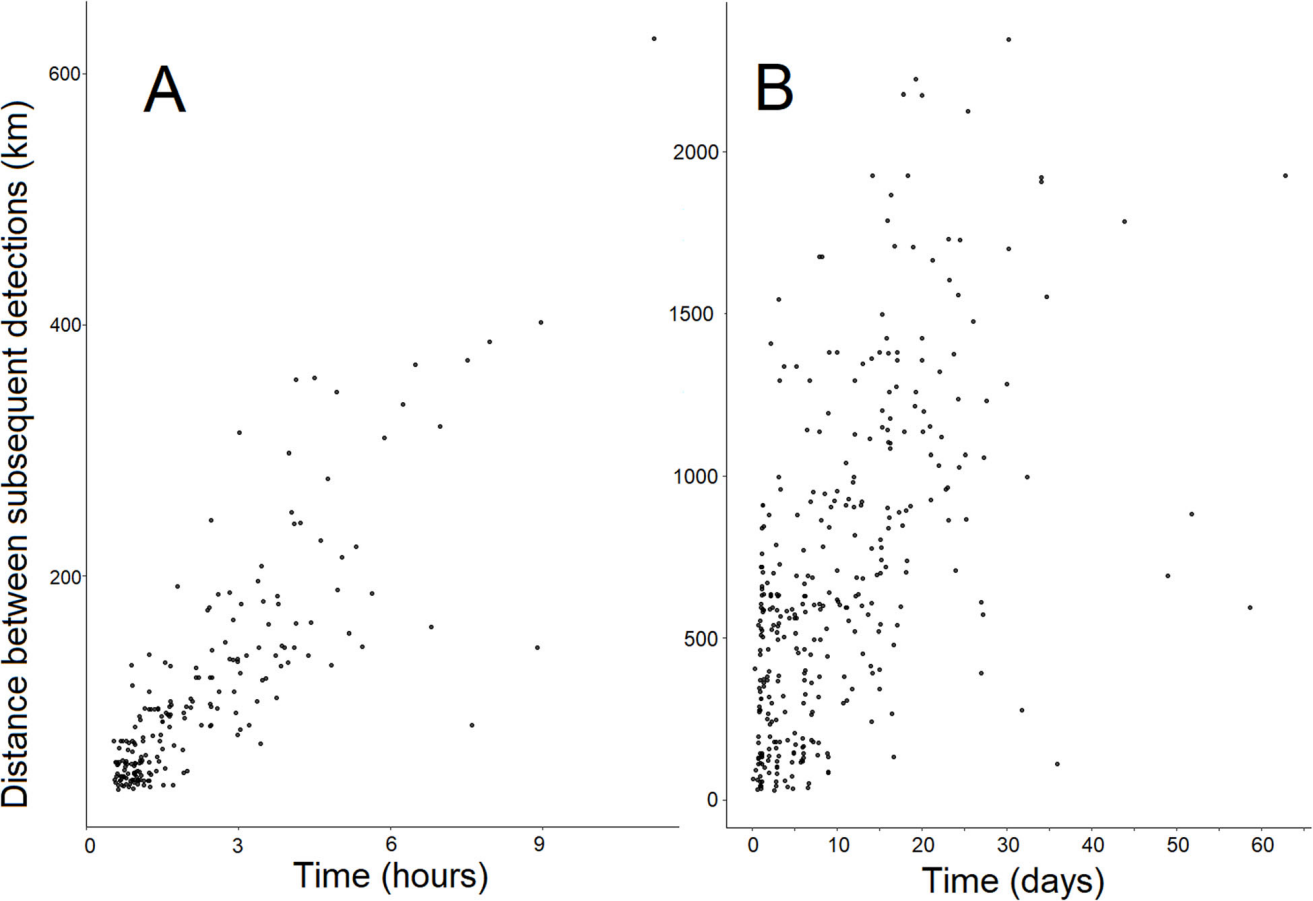
Time (**a**: hours, **b**: days) between successive detections in relation to distance between stations (km)) and migration pace (km/h) of 553 segments from 236 Swainson’s Thrushes for segments within the same day (**a**) and > 1 day (**b**). Migration pace is the result of the distance and the time elapsed between two receiving stations. The migration pace does not indicate the ground speed of the birds as the distance between receiving stations is not representative of the distance traveled by the bird

Of the 241 individuals detected during fall migration, 5 individuals had segments < 30 km and/or < 30 min and were removed from the migration pace analysis. Hence, the radio-telemetry network recorded 552 segments (i.e. sequential detections between two receiving stations located a minimum of 30 km and 30 min apart) from 236 individuals, including 107 juveniles and 129 adult Swainson’s Thrushes. Migration pace ranged between 0.13 and 146.2 km/h (23 ± 24.4 km/h [Mean ± SD]). We detected 209 segments occurring on the same day (Fig. 3a), including one daytime segment (mean = 53.3 km/h, range = 10.7–146.2 km/h); and 343 segments more than one day apart (Fig. 3b, mean = 6 km/h, range = 0.13–62.6 km/h).

**Fig. 4:**
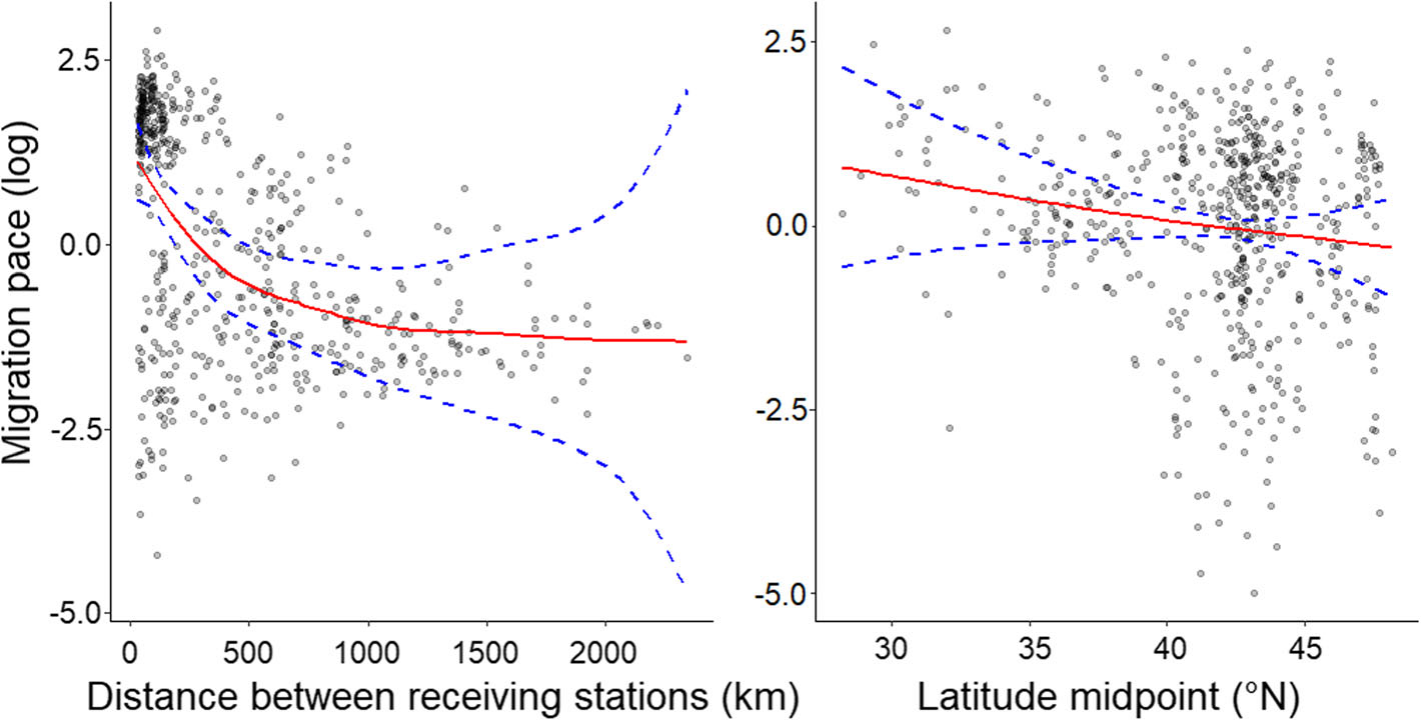
Fitted splines for a generalized additive model of the relationship between distance between receiving stations and latitude (smooth terms) on migration pace (log). Distance between receiving stations (left) suggest a lack of variation of the migration pace for longer segments. Birds have a slower migration pace in northern latitude, closer to their breeding origin (right). Migration paces were slower and more variable in northern latitudes suggesting more stopover closer to the breeding grounds

We evaluated four generalized additive mixed models (GAMM) to test the influence of tagging longitude, age and the interaction between age and latitude on migration pace (log), including distance between receiving stations as a covariate and year and bird ID as random effects (*n* = 552). The full model explained 25.4% of the deviance (*R*^2^ adjusted = 0.24). After performing multi-model inference on all models, age, the interaction between age and latitude, and tagging longitude had no significant effect on migration pace. Migration pace decreased swiftly with increasing distance between receiving stations for short segments distances (< 1000 km) and leveled off for longer segments distances (− 1.26 ± 0.5, *p* = 0.02, Fig. 4), suggesting a lack of variation of migration pace for longer segments. Opposite to our prediction, when controlling for the distance between receiving stations, birds migrated more slowly at more northern latitudes, i.e. closer to their breeding range (− 0.21 ± 0.1, *p* = 0.03, Fig. 4).

## Discussion

Despite converging through the same area north of the Gulf of Mexico, Swainson’s thrush populations from across eastern Canada tended to maintain population specific migration routes. While we expected a decrease of migratory connectivity due to a general convergence of birds north of the Gulf of Mexico given presumed migration routes, we did not expect to find local spatial structure within migration routes in and around Florida. This finding is important, as it provides evidence for a certain degree of local spatial structure despite regional convergence in migratory songbird sub-populations.

Broadly, the birds from the easternmost tagging site (ABO) migrated along the Atlantic coast while the westernmost individuals (BPBO) adopted an inland route and were not detected near coastal receiving stations. Interestingly, given the absence of detections along the coast of Texas and Louisiana, most birds likely undertook an overwater flight toward the Yucatán peninsula or the Caribbean rather than a route following the west coast of the Gulf of Mexico as observed in western populations [16, 46, 47]. Unfortunately, the Motus network array did not cover the coast of Georgia and Alabama and thus, we do not know whether some birds crossed the Gulf of Mexico further west.

We found evidence that migrating Swainson’s thrushes maintained population spatial structure near the Florida Peninsula, however, evidence for this structure varied depending on the connectivity analysis carried out. The connectivity analysis based on distances between locations (e.g., [14, 29, 50]) suggested a lack of spatial structure, whereas our analysis of migratory connectivity *en route* (e.g., [5]) provides evidence of spatial structure. The latter results was also supported by our multivariate analysis, which showed finer scale spatial structure along latitude but not longitude around the Florida Peninsula. Had we focused our analysis on a distance based measure of migratory connectivity only, we would have missed the latitudinal structure observed around Florida. We suggest that the suite of methods that we used in this study to assess connectivity are complimentary and help paint a more complete picture of how migratory connectivity can vary along migration routes.

While we found evidence for spatial and temporal migratory connectivity around the Florida Peninsula, despite regional convergence, we did not investigate the drivers of the local-scale spatial and temporal segregation that we observed. Differences in migratory spatial and temporal migratory connectivity may be driven by both intrinsic differences among populations in the timing of migration and migration routes as well as by differences in extrinsic weather conditions experienced along the migration route. For example, wind conditions aloft can drive the pace of migration [39] and stopover durations, particularly at ecological barriers [18]. Winds can also influence migration tracks and thus routes [25]. Differences in migration routes could also be driven by the age structure of the populations being tagged [6, 15]. For example, juveniles can have different intrinsic migration routes or respond differently to wind conditions aloft [39], which would likely decrease the strength of migratory connectivity. We suggest examining the relative importance of intrinsic and extrinsic drivers of migratory connectivity is an important area of future research. An important caveat of our results is that in our study, except for individuals captured directly on their breeding site (ABO, FM, MV), the breeding origin of tagged birds could not be established. However, we believe that breeding origin had little impact on migratory connectivity between early and later stages of the migration journey.

Migration paces for individuals were slower at more northern latitudes. Birds likely stayed stationary or made non linear displacements between receiving stations, such as landscape-scale movements within stopovers (e.g. foraging or exploratory movements) [38, 53]. The Great Lakes basin, the Gulf of Maine and the Atlantic Coast areas are all known to concentrate migratory songbirds during migration, and stopovers are known to occur in these regions, as birds determine how to navigate these potential ecological barriers [7, 34, 41]. Therefore, the slow migration pace exhibited in these areas likely reflect stopover activity. Alternatively, the slower migration paces observed at more northern latitudes might represent a gradual transition and increase in movement as birds transition from post-fledging movement and activities to full migratory movements. Swainson’s Thrushes perform longer or more frequent stopovers in the southern part of their migration route [5]. In our study, we included distance as a covariate to limit potential biases, yet the density of the radio-telemetry network still had a strong influence on the accuracy of the calculated migration pace. A denser radio-telemetry array enables researchers to distinguish between sustained flights and extended resting periods more readily. In other words, the longer distances between receiving stations reduced our ability to estimate migration pace and detect stopovers with confidence. We highlight an important gap in the Motus network between southern Virginia and northern Florida, which did not allow us to determine stopover activity. Western “inland” Swainson’s Thrushes monitored with geolocators refueled north of the Gulf of Mexico before crossing the Gulf of Mexico [16]. In our study, most birds detected near the Florida peninsula exhibited a clear flight pattern with high migration pace suggesting that there were flying by, or that unlike the Western “inland” population of Swainson’s Thrushes, they might not stop north of the Gulf.

We found no evidence for a relationship between age and migration pace, although other studies found differences in migration pace between adults and juveniles closer to the breeding sites [6, 39]. Our study captured migratory movements away from the breeding sites and it is likely that age differences are less important as birds progress south and gain experience. Age related differences may also vary between species given different selection pressures on the pace of migration. Similar to age, we found that tagging longitude did not influence migration pace. Although they were captured *en route*, individuals tagged at OOT likely originated from the Quebec-Labrador peninsula [19], but individuals captured at more southern migratory sites, like BPBO or MBO, may have originated from a wider region [24]. Other factors including moult status, sex, body condition, wing morphology might have influenced the migration pace [9, 17, 40], but they were not further investigated in this study.

The objective of our study was not to estimate the migration ground speed of Swainson’s Thrushes. However, we noted a considerable proportion of migration paces calculated for segments occurring on the same day were > 43 km/h (75 p.c. = 43.12 km/h). The high migration paces calculated for segments occurring on the same day might result from an overestimation of the distance between detection due to the detection range of the receiving stations, the presence of strong winds or tropospheric propagation [12]. Nevertheless, the high migration paces calculated for segments occurring on the same day are similar to the maximum ~ 80 km/h previously estimated for migrating Swainson’s Thrushes with manual radio-telemetry [13]. We recognize that the radio-telemetry array had temporal and spatial gaps between receiving stations. Nevertheless, the migration paces calculated from the raw data suggest that migrating Swainson’s Thrushes have the capacity to travel much more than 100 km/day, contrarily to what was suggested by previous studies for fall migration (i.e. 100–120 km/day) [57]. Yet, one individual Swainson’s Thrush previously monitored in spring with radio-telemetry traveled up to 375 km/night [13]. Moreover, Gómez et al. [22] estimated the flight range of Swainson’s Thrush to be approximately 680–800 km after a fall stopover in Colombia based on fat deposition and fuel load, although this does not account for headwinds that could be encountered during flight. None the less, the large migration distances observed and the high migration paces observed do suggest that Swainson’s Thrushes rely on fewer resting and stopover areas than previously expected to complete their fall migration. It is therefore important to identify the exact locations of stopover sites to evaluate the habitat used like conifer or deciduous forest patches [33] and needed during migration and to gain a better understanding of the time required to refuel to understand the migratory strategy of Swainson’s Thrush.

The Motus Wildlife tracking system is an accessible and effective technology to gather data across a species breeding range. The receiving array covered adequately the northern part of the coastal plains (Embayed section) and allowed for more accurate tracking of coastal populations like the birds originating from the Maritimes. The southern part of regions including Piedmont, Blue Ridge and the Appalachian Plateaus, and East Gulf coastal plains would require supplementary receiving stations to track more inland migration routes. Reducing distance between receiving stations by developing of more receiving stations within the radio-telemetry array would reduce the bias of distance and enhance the accurate estimation of the migration pace.

## Conclusion

In summary, our study used birds tagged across the eastern breeding / migration range (a 1200 km gradient) and provided the first assessment of migratory connectivity during the fall migration period for the eastern populations of Swainson’s thrush. Importantly, we found that at a broad scale, migratory connectivity decreased and birds converged geographically as they migrated south. However, despite a weaker connectivity, we show for the first time that a population of migratory birds still appeared to maintain finer-scale spatial structure in their migration routes in a zone of convergence, suggesting that conservation strategies for different breeding populations of migratory birds may need to consider fine-scale migration routes in the convergence zone. Our approach provides a framework for understanding differences in migration routes among populations based on detections in the Motus Wildlife Tracking System and will ultimately allow for an improved understanding the factors driving migration patterns in Swainson’s Thrush and other species. We hope that our results will encourage additional deployments of receiving stations in the Motus radio-telemetry network to fill important geographic gaps and more collaboration among the different research projects to achieve a more complete portrait of the migration phenomenon.

## Supplementary Information

**Additional file 1.**

## Data Availability

The datasets used and/or analysed during the current study are available from the corresponding author on reasonable request.
