# Supplementary material for: Spatial structure in migration routes maintained despite regional convergence among eastern populations of Swainson’s Thrushes
Source: Mov Ecol. 2021 May 13;9:23. doi: 10.1186/s40462-021-00263-9 (PMC8117314; doi:10.1186/s40462-021-00263-9)

**TITLE**

Spatial structure in migration routes maintained despite regional convergence among eastern populations of Swainson's Thrushes

**AUTHORS**

Bégin Marchand, Camille[1]*, Desrochers, André[2], Taylor, Philip D.[3,4], Tremblay, Junior A.[1,2], Berrigan, Lucas[4], Frei, Barbara[6,7], Morales, Ana[4], Mitchell, Greg W.[8,9]

*Corresponding author

[1] Wildlife Research Division, Environment and Climate Change Canada, 1550 d'Estimauville, Québec, QC. G1J 0C3 Canada

[2] Université Laval, 2325 Rue de l'Université, Québec, QC. G1V 0A6 Canada

[3] Acadia University, 33 Westwood Avenue, Wolfville, NS. B4P 2R6 Canada

[4] Birds Canada, P.O. Box 6227, 17 Waterfowl Lane, Sackville, NB. E4L 1G6 Canada

[5] McGill University, 21111 Lakeshore Road, Ste. Anne de Bellevue, QC. H9X 3V9 Canada

[6] McGill Bird Observatory, The Migration Research Foundation, Inc., PO Box 10005, Ste Anne de Bellevue, QC, H9X 0A6 Canada

[7] Canadian Wildlife Service, Environment and Climate Change Canada, 351 boul. Saint-Joseph, Gatineau, QC, K1A 0H3 Canada

[8] Wildlife Research Division, Environment and Climate Change Canada, 1125 Colonel By Rd., Ottawa, ON, K1A 0H3, Canada

[9] Department of Biology, Carleton University, 1125 Colonel By Dr., Ottawa, ON, K1S 5B6, Canada

**SUPPLEMENTARY MATERIAL**

**Table S1.** Parameters of the 16 intervals of 5°N latitude: cell size in degree (1 x 1, 0.1 x 0.1, 0.01 x 0.01), $R^M$ coefficients (partial Mantel test, 10 000 permutations), number of individuals (n

Birds) and receiving stations (N stations) per interval, standard error and lower/upper limit of the 95% confidence interval based on 100 bootstraps samples.

| Cell size (degree) | Interval (°N) | N Birds | N Stations | R Mantel | Standard error | 95% CI Lower limit | 95% CI Upper limit |
|---|---|---|---|---|---|---|---|
| 1 x 1 | 45 to 40 | 142 | 36 | 0,35 | 0,05 | 0,26 | 0,44 |
| 1 x 1 | 44 to 39 | 186 | 40 | 0,28 | 0,04 | 0,20 | 0,35 |
| 1 x 1 | 43 to 38 | 174 | 35 | 0,26 | 0,04 | 0,18 | 0,33 |
| 1 x 1 | 42 to 37 | 155 | 28 | 0,21 | 0,04 | 0,14 | 0,28 |
| 1 x 1 | 41 to 36 | 126 | 22 | 0,13 | 0,03 | 0,07 | 0,20 |
| 1 x 1 | 40 to 35 | 95 | 14 | 0,12 | 0,04 | 0,04 | 0,21 |
| 1 x 1 | 39 to 34 | 24 | 8 | 0,27 | 0,14 | 0,00 | 0,54 |
| 1 x 1 | 38 to 33 | 21 | 7 | 0,36 | 0,17 | 0,04 | 0,69 |
| 1 x 1 | 37 to 32 | 27 | 6 | 0,30 | 0,10 | 0,10 | 0,49 |
| 1 x 1 | 36 to 31 | 21 | 5 | 0,39 | 0,21 | -0,02 | 0,80 |
| 1 x 1 | 35 to 30 | 34 | 7 | 0,14 | 0,08 | 0,00 | 0,29 |
| 1 x 1 | 34 to 29 | 63 | 11 | 0,18 | 0,04 | 0,10 | 0,27 |
| 1 x 1 | 33 to 28 | 68 | 10 | 0,15 | 0,04 | 0,07 | 0,23 |
| 1 x 1 | 32 to 27 | 65 | 10 | 0,17 | 0,04 | 0,08 | 0,25 |
| 1 x 1 | 31 to 26 | 65 | 9 | 0,17 | 0,05 | 0,07 | 0,26 |
| 1 x 1 | 30 to 25 | 59 | 8 | 0,15 | 0,06 | 0,04 | 0,27 |
| 0.1 x0.1 | 45 to 40 | 142 | 125 | 0,25 | 0,03 | 0,18 | 0,31 |
| 0.1 x0.1 | 44 to 39 | 186 | 141 | 0,22 | 0,03 | 0,17 | 0,28 |
| 0.1 x0.1 | 43 to 38 | 174 | 122 | 0,20 | 0,03 | 0,15 | 0,26 |
| 0.1 x0.1 | 42 to 37 | 155 | 81 | 0,20 | 0,03 | 0,13 | 0,26 |
| 0.1 x0.1 | 41 to 36 | 126 | 63 | 0,14 | 0,03 | 0,08 | 0,20 |
| 0.1 x0.1 | 40 to 35 | 95 | 36 | 0,16 | 0,04 | 0,08 | 0,24 |
| 0.1 x0.1 | 39 to 34 | 24 | 17 | 0,22 | 0,13 | -0,03 | 0,46 |
| 0.1 x0.1 | 38 to 33 | 21 | 12 | 0,20 | 0,18 | -0,16 | 0,56 |
| 0.1 x0.1 | 37 to 32 | 27 | 9 | 0,24 | 0,10 | 0,03 | 0,44 |
| 0.1 x0.1 | 36 to 31 | 21 | 7 | 0,37 | 0,19 | -0,02 | 0,75 |
| 0.1 x0.1 | 35 to 30 | 34 | 11 | 0,21 | 0,08 | 0,05 | 0,37 |
| 0.1 x0.1 | 34 to 29 | 63 | 21 | 0,18 | 0,04 | 0,10 | 0,27 |
| 0.1 x0.1 | 33 to 28 | 68 | 22 | 0,15 | 0,03 | 0,09 | 0,22 |
| 0.1 x0.1 | 32 to 27 | 65 | 22 | 0,17 | 0,04 | 0,08 | 0,25 |
| 0.1 x0.1 | 31 to 26 | 65 | 19 | 0,16 | 0,04 | 0,08 | 0,25 |
| 0.1 x0.1 | 30 to 25 | 59 | 17 | 0,15 | 0,07 | 0,02 | 0,28 |
| 0.01 x 0.01 | 45 to 40 | 142 | 141 | 0,25 | 0,03 | 0,20 | 0,31 |
| 0.01 x 0.01 | 44 to 39 | 186 | 163 | 0,25 | 0,03 | 0,20 | 0,31 |
| 0.01 x 0.01 | 43 to 38 | 174 | 145 | 0,25 | 0,02 | 0,20 | 0,29 |
| 0.01 x 0.01 | 42 to 37 | 155 | 99 | 0,24 | 0,03 | 0,19 | 0,30 |
| 0.01 x 0.01 | 41 to 36 | 126 | 76 | 0,20 | 0,03 | 0,15 | 0,25 |

| | | | | | | | |
|---|---|---|---|---|---|---|---|
| 0.01 x 0.01 | 40 to 35 | 95 | 44 | 0,25 | 0,03 | 0,18 | 0,32 |
| 0.01 x 0.01 | 39 to 34 | 24 | 19 | 0,18 | 0,13 | -0,07 | 0,43 |
| 0.01 x 0.01 | 38 to 33 | 21 | 12 | 0,20 | 0,16 | -0,11 | 0,50 |
| 0.01 x 0.01 | 37 to 32 | 27 | 10 | 0,35 | 0,10 | 0,15 | 0,54 |
| 0.01 x 0.01 | 36 to 31 | 21 | 8 | 0,51 | 0,18 | 0,17 | 0,86 |
| 0.01 x 0.01 | 35 to 30 | 34 | 13 | 0,36 | 0,09 | 0,18 | 0,54 |
| 0.01 x 0.01 | 34 to 29 | 63 | 24 | 0,21 | 0,04 | 0,12 | 0,30 |
| 0.01 x 0.01 | 33 to 28 | 68 | 25 | 0,19 | 0,05 | 0,09 | 0,28 |
| 0.01 x 0.01 | 32 to 27 | 65 | 24 | 0,20 | 0,05 | 0,10 | 0,29 |
| 0.01 x 0.01 | 31 to 26 | 65 | 21 | 0,19 | 0,05 | 0,10 | 0,28 |
| 0.01 x 0.01 | 30 to 25 | 59 | 18 | 0,15 | 0,05 | 0,04 | 0,26 |

**Table S2.** Description of the different tag models deployed among the different tagging site and year of capture: burst interval, estimated lifespan of all tags deployed, number of tags deployed and approximate starting and ending dates associated. We used the median deployment date for the starting date because we deployed tags on different days within a tagging season.

| Site | Year | Burst interval | lifespan | N | Starting date (median) | Ending date |
|---|---|---|---|---|---|---|
| ABO | 2016 | 11 | 198 | 26 | 2016-08-07 | 2017-02-21 |
| ABO | 2016 | 11 | 390 | 14 | 2016-08-29 | 2017-09-23 |
| ABO | 2016 | 23 | 266 | 1 | 2016-08-03 | 2017-04-26 |
| ABO | 2016 | 35 | 303 | 5 | 2016-09-02 | 2017-07-02 |
| ABO | 2017 | 11 | 390 | 1 | 2017-09-06 | 2018-10-01 |
| ABO | 2017 | 20 | 515 | 61 | 2017-08-20 | 2019-01-17 |
| BPBO | 2017 | 20 | 515 | 25 | 2017-09-14 | 2019-02-11 |
| BPBO | 2018 | 20 | 445 | 24 | 2018-09-12 | 2019-12-01 |
| FM | 2014 | 13 | 213 | 15 | 2014-07-17 | 2015-02-15 |
| FM | 2015 | 10 | 186 | 5 | 2015-06-25 | 2015-12-28 |
| FM | 2015 | 13 | 209 | 8 | 2015-06-11 | 2016-01-06 |
| FM | 2016 | 5 | 254 | 13 | 2016-06-08 | 2017-02-17 |
| FM | 2017 | 20 | 515 | 8 | 2017-07-25 | 2018-12-22 |
| MBO | 2017 | 5 | 233 | 2 | 2017-07-29 | 2018-03-19 |
| MBO | 2017 | 6 | 134 | 20 | 2017-08-11 | 2017-12-23 |
| MBO | 2018 | 7 | 212 | 38 | 2018-08-12 | 2019-03-12 |
| MBO | 2018 | 10 | 79 | 10 | 2018-09-15 | 2018-12-03 |
| MBO | 2018 | 15 | 159 | 6 | 2018-09-28 | 2019-03-06 |

| | | | | | | |
|---|---|---|---|---|---|---|
| MV | 2017 | 20 | 515 | 8 | 2017-08-08 | 2019-01-05 |
| OOT | 2014 | 6 | 137 | 36 | 2014-09-19 | 2015-02-03 |
| OOT | 2014 | 13 | 213 | 6 | 2014-09-15 | 2015-04-16 |
| OOT | 2015 | 10 | 186 | 3 | 2015-09-15 | 2016-03-19 |
| OOT | 2015 | 13 | 209 | 23 | 2015-09-24 | 2016-04-20 |
| OOT | 2016 | 5 | 254 | 1 | 2016-09-24 | 2017-06-05 |
| OOT | 2016 | 11 | 390 | 15 | 2016-09-12 | 2017-10-07 |
| OOT | 2017 | 20 | 515 | 18 | 2017-09-15 | 2019-02-12 |

Table S3.

**Figure S1.** Migration rates (km/h) calculated for 210 segments occuring on the same night for 110 Swainson's Thrushes equiped with radio-transmitters. Migration rates were calculated by mesuring the distance between receving stations and the time elapsed between consecutive detections. The migration rates do not represent the ground speed of migrating thushes as distance might have been overestimated due to the variation of the detection range of the receiving stations. The mean migration rate (50.3 km/h) is represented by the vertical broken line.

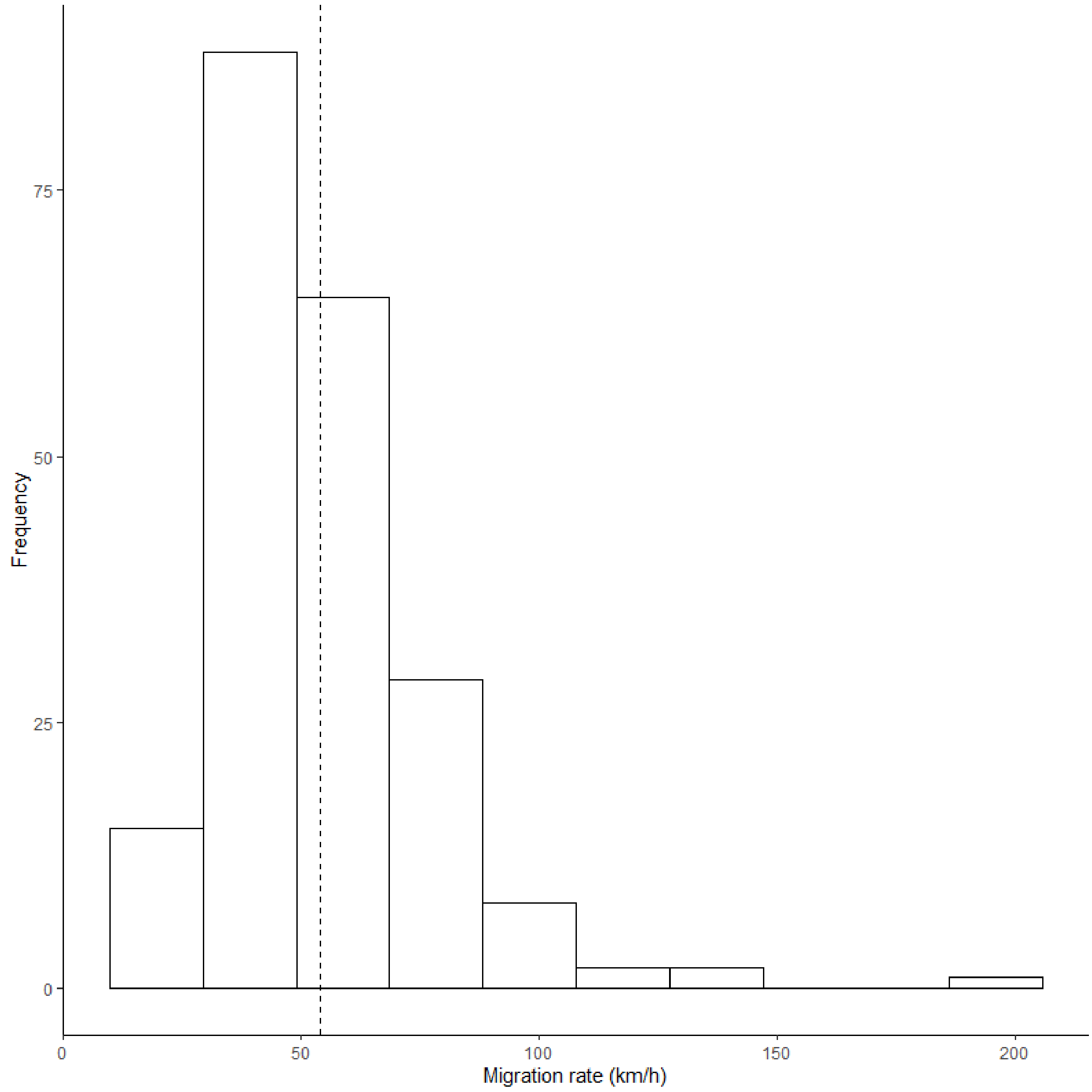

Supplement: Supplementary file 1 — Additional file 1. [file 40462_2021_263_MOESM1_ESM.pdf]
